# Eosinopenia as a marker of sepsis and mortality in critically ill patients

**DOI:** 10.1186/cc14127

**Published:** 2015-03-16

**Authors:** A Savitskiy, V Rudnov, V Bagin

**Affiliations:** 1 City Clinical Hospital # 40, Yekaterinburg, Russia

## Introduction

The idea of using the eosinophil count (EC) as a diagnostic marker for clarifying the nature of systemic inflammatory response syndrome (SIRS) belongs to K Abidi, who showed that EC could be used as a diagnostic criterion of sepsis. There are no published data to define the role of dynamic control of EC in the process of intensive therapy as a prognostic marker and indicator of severity condition in critically ill patients. The aim was to determine the informative value of EC in the development of SIRS as a biomarker of sepsis and indicator of the severity condition and prognosis of outcome in the pathological process.

## Methods

A total of 143 patients were enrolled in this study who were admitted to the ICU and had SIRS. All patients were divided into a septic group - patients with community-acquired pneumonia, complicated by sepsis - and two SIRS groups of noninfectious genesis - patients who had an acute cerebrovascular accident (CVA) and an acute myocardial infarction (AMI). The absolute EC was measured at admission and in the dynamics on days 3 to 5 of stay.

## Results

The median EC was 75 cells/mm^3 ^in septic patients on admission, which was significantly lower than in patients with CVA (120 cells/mm^3^) and AMI (130 cells/mm^3^). Comparison of EC in septic patients between survivors and those who died showed significant differences (Table 1). Receiver operating characteristic (ROC) analysis determined a value less than 80 cells/mm^3 ^as the optimal diagnostic cutoffvalue with a high level of confidence in the comparison of septic and noninfectious groups. Area under the ROC curves was 0.94, sensitivity of 80.8%, specificity of 95.6%, *P *< 0.0001. There was a significant increase of EC in survivors, while the EC did not change significantly among those who died in the dynamics. ROC analysis determined the cutoff values of EC, which indicated a high risk of an adverse outcome in septic patients (Table 2).

**Table 1:** Dynamics of eosinophil count depending on outcome in groups.

	EC (cells/mm^3^) at admission			EC (cells/mm^3^) on the 3rd to 5th days		
	
Group	Survivors	Died	*P *value	Survivors	Died	*P *value
Sepsis	100	50	0.006	240	70	0.0004
CVA	120	115	0.74	150	90	0.001
AMI	145	120	0.60	195	120	0.017

**Table 2:** Informational value of eosinophil count in assessment of disease outcome.

Day/Group	AUC	Cutoff (EC, cells/mm^3^)	Sensitivity (%)	Specificity (%)	*P *value
At admission					
Sepsis	0.83	≤220	100	61.5	0.0001
CVA	0.53	≤150	78.3	37.5	0.743
AMI	0.56	≤190	85.7	35.7	0.6136
On the 3rd to 5th days					
Sepsis	0.91	≤120	92.3	69.2	<0.0001
CVA	0.81	≤140	91.3	68.8	<0.0001
AMI	0.75	≤90	92.9	46.7	0.0052

## Conclusion

EC may be an additional diagnostic marker which characterizes the nature of SIRS. Eosinopenia associated with prognosis of outcome in critical conditions.

